# Patient Satisfaction after Laparoscopic Nissen Fundoplication—Long-Term Outcomes of Single-Center Study

**DOI:** 10.3390/jcm10245924

**Published:** 2021-12-17

**Authors:** Natalia Dowgiałło-Gornowicz, Justyna Kacperczyk, Anna Masiewicz, Paweł Lech, Sławomir Saluk, Karolina Osowiecka, Maciej Michalik

**Affiliations:** 1 Department of General, Minimally Invasive and Elderly Surgery, Collegium Medicum, University of Warmia and Mazury, Niepodległosci 44 St., 10-045 Olsztyn, Poland; justyna.kacperczyk@gmail.com (J.K.); annalukuc@gmail.com (A.M.); lechpawel@op.pl (P.L.); slawomirsaluk@gmail.com (S.S.); 2 Department of Psychology and Sociology of Health and Public Health, School of Public Health, University of Warmia and Mazury in Olsztyn, Warszawska 30 St., 11-041 Olsztyn, Poland; k.osowiecka86@gmail.com; 3 Department of General, Colorectal and Oncologic Surgery, Collegium Medicum, Nicolaus Copernicus University in Torun, Ujejskiego 75 St., 85-168 Bydgoszcz, Poland; michs1@wp.pl

**Keywords:** laparoscopic Nissen fundoplication, GERD, gastroesophageal reflux disease, patient satisfaction

## Abstract

Up to 33% of the population suffers from gastroesophageal reflux disease (GERD). Given its high prevalence, the negative impact on quality of life, and the possible progression to esophageal cancer, the definitive treatment of GERD should be used more frequently. This study aims to assess long-term patient satisfaction after laparoscopic Nissen fundoplication (LNF). We reviewed the prospectively collected data of patients who underwent LNF for GERD in our department in 2014–2018. Each patient completed a preoperative questionnaire according to GERD Impact Scale (GERD-IS). Postoperative survey consisted of GERD-IS, the need for PPIs, and two “yes or no” questions to assess satisfaction with the outcome. The mean follow-up time was 50 months (21.2–76.3 ± 16.6 months). There was a statistically significant improvement in each GERD-IS question (*p* < 0.001). A total of 87 patients (78.4%) would recommend the surgery to their relatives. Patients without symptom recurrence and without the need for chronic PPI use after surgery were significantly more likely to recommend surgery and to undergo the procedure again (*p* < 001). The age of patients did not influence patients’ recommendations (*p* = 0.75). A total of 17 patients (15.3%) would not undergo LNF again. There was no significant correlation between the answer and patient’s complications or age (*p* > 0.05). LNF is a good treatment for GERD with a satisfaction rate of 78.4%.

## 1. Introduction

Up to 33% of the adult population suffers from gastroesophageal reflux disease (GERD), which is caused by the reflux of gastric contents into the esophagus [1]. It is associated with disturbing symptoms such as heartburn, nausea, or epigastric pain. Patients may also suffer from extraesophageal symptoms such as a cough [2]. Due to the high prevalence of the disease, the negative impact on patients’ quality of life and possible progression to esophageal cancer, definitive treatment of GERD should be used more frequently.

Treatment for GERD involves the use of medicaments as proton pump inhibitors (PPIs), which can relieve symptoms but do not cure the cause of GERD. Therefore, the treatment for GERD is anti-reflux surgery, which is becoming the standard [3]. The most common procedure is laparoscopic Nissen fundoplication (LNF) [4]. Despite the well-described advantages of surgical treatment, it still does not meet the needs of the population. The latest data from Korea show that, of the 3.1 million patients undergoing PPIs therapy, only 342 underwent surgery between 2012 and 2016 [4]. The above data show that only 1% of the population with GERD underwent surgery. It can be assumed that this is due to the lack of informing family doctors who first contact the patients, or the patients’ fear of the course of the surgery, possible complications, and long-term outcomes [5]. Therefore, emphasis should be placed on patient satisfaction after the surgery as well as on its outcomes, including the need for PPIs or symptom relief.

This study aims to assess the long-term patient satisfaction after LNF, especially whether they would undergo the procedure again or recommend it to their relatives.

## 2. Materials and Methods

We reviewed prospectively collected data of patients who underwent LNF for GERD in our department from 2014 to 2018 by the same team of surgeons. Prior to surgery, all patients underwent objective studies to establish the diagnosis of GERD, including esophagogastroduodenoscopy, 24 h pH measurement study, and otorhinolaryngological examination. Each patient completed a preoperative questionnaire for their demographics, preoperative examinations, and symptoms according to GERD Impact Scale (GERD-IS). Then, it was registered in the database. The postoperative survey was performed during personal, or telephone consultations carried out one month after the surgery and every twelve months thereafter. The questionnaire included the actual GERD-IS and questions regarding the recurrence of symptoms and the need for PPIs. Additionally, it consisted of two “yes or no” questions to assess satisfaction with the result: would they recommend the surgery to their relatives and whether they would undergo the procedure again with the knowledge they have now. As patients avoid a definite answer, the number of patients studied varied depending on these two questions. Each patient signed the appropriate consent to participate in the study, along with the consent of our institution to conduct the study. Due to study design, it was not formally supervised by an Institutional Review Board in line with its policy.

All patients underwent LNF according to standard technique. After dissection of the crura of the diaphragm, the distal esophagus was mobilized approximately 5 cm. The crura were sewn behind the esophagus with 2–3 non-absorbable sutures. A posterior 360° wrap of the fundus, approximately 2 cm long, was constructed with 2–3 non-absorbable sutures.

### Statistical Analysis

Descriptive statistics were used. The median, first quartile (Q1), and the third quartile (Q3) of the GERD-IS distribution were estimated. Differences in GERD-IS before and after surgery were determined using Wilcoxon test. The Mann–Whitney test was performed to compare the medians due to various factors. The proportion in the analyzed subgroups was compared using the chi-square test. A *p* value < 0.05 was considered statistically significant. The analysis was conducted using TIBCO Software Inc., Krakow, Poland (2017) Statistica (data analysis software system), version 13.

## 3. Results

In total, 111 patients (49 female, 62 male) were analyzed in the study. All patients underwent LNF in one surgical department in 2014–2018 by the same team of surgeons. The mean age was 50.2 years (18–80 years, ±15 years) and the mean body mass index was 25.9 kg/m^2^ (17.7–35.6 kg/m^2^ ± 3.5 kg/m^2^). The mean follow-up time was 50 months (21.2–76.3 ± 16.6 months) (Table 1). Each patient had an endoscopic examination before the operation. Of these, 14 (12.6%) patients had a diagnosis of hiatal hernia without esophagitis, and 97 patients (87.4%) had esophagitis. Esophagitis was described according to the Los Angeles (LA) Classification. The most numerous was LA-A, described in 62 patients (63.9%), while LA-D was seen in two cases (2.1%) (Table 1). None of the patients showed Barret’s esophagus in the preoperative gastroscopy. Overall, 25 (22.5%) patients had arterial hypertension, and 8 (7.2%) patients had type 2 diabetes. For this reason, they were taking oral medications. All patients took PPIs in the period prior to surgery. No patient was taking psychiatric medications. There were five early postoperative complications and no conversion to open surgery. One patient, a 68-year-old woman with acute respiratory failure, was transferred to the intensive care unit and after two days returned to the surgery department and was discharged on the fifth postoperative day in a good condition. One patient underwent splenectomy during the initial surgery due to their injury. However, this did not extend the length of stay. Three patients required reoperation in the first month after surgery: two due to too tight fundoplication wrap and one due to early recurrence. Seventeen patients (15.3%) required chronic PPIs administration (more than one month after surgery); recurrence of symptoms occurred in 24 patients (21.6%) on average 11.9 months (0–24 ± 5.5 months) after LNF. In follow-up, six patients reported chronic gas bloating syndrome after surgery, which did not require medication, but only dietary modification. Two patients reported mild dysphagia which also required diet modification.

All patients answered the GERD-IS questionnaire at the last follow up visit. There was a statistically significant improvement in symptoms in every of the nine GERD-IS questions (*p* < 0.001), Table 2.

At the last follow-up visit, patients also answered two “yes or no” questions. Not every patient who answered two questions determined their satisfaction with the operation. In the first—if they recommend LNF to their relatives—102 patients were sure of the answers, 8% were unanswered; 87 of those who answered (78.4%) would recommend surgery. The age of patients did not influence the patient recommendations (*p* = 0.75). Half of the patients without complications would recommend surgery (*p* = 0.04). Patients without recurrence of symptoms and without the need for chronic PPI use after surgery stated that they would recommend surgery to their relatives significantly more often (*p* < 001) (Table 3).

The second question was whether, with the knowledge they have now, they would undergo surgery again. In this case, more patients answered this question unequivocally than in the previous one (105 patients); 17 patients (15.3%) would not undergo LNF again. Patients without recurrence of symptoms and without the need for chronic PPI use after surgery were significantly more likely to be open to undergoing the surgery again (*p* < 0.001). There was no significant correlation between the answer and patient’s complications or age (*p* > 0.05), Table 3.

All 15 patients who would not recommend LNF to their relatives and 17 (15 from the first group + 2 more) patients who would not wish to undergo LNF again were identified as dissatisfied and asked for their rationale. Two of them, after early stenosis, were not satisfied with the need for reoperation and 12 had recurrence of symptoms or needed PPIs and did not think about the surgery as a cure. Three patients, despite their symptom relief, would not wish to undergo perioperative stress again.

## 4. Discussion

This prospectively collected review of patient satisfaction after LNF confirms that this anti-reflux surgery is a good treatment for GERD. To our knowledge, this issue concerns one of the largest groups of patients with a long-term follow-up and one of the first in Central Europe. The value of the work is increased by the fact that previous papers were published 7–20 years ago, and knowledge about anti-reflux surgery changed during this period [6,7,8,9,10]. Therefore, it was necessary to update the data. 

GERD is a serious problem for the European population and has serious psychological and social consequences. It affects about 20% of people and, if left untreated, has a negative impact on quality of life [1,4]. Therefore, complete and permanent symptom relief has become an important point in the search for effective treatment. It is said that we have two types of symptoms—typical and extraesophageal. Although the typical symptoms can be eliminated with drugs, only surgical methods can cure permanently [11,12,13,14]. Nevertheless, not all patients benefit from anti-reflux surgery, so selecting patients for LNH remains a challenging task.

All patients operated on in our department underwent LFN. LFN has been described as a good treatment for GERD patients [15,16,17]. However, there are other surgical anti-reflux treatments as well. Laparoscopic Toupet fundoplication is also performed with a similar frequency. By comparing the early and late results, both procedures achieve similar results according to the available meta-analyzes [16,17]. Therefore, the choice of surgery should be based on the surgeon’s experience and patient involvement.

Overall, 78.4% of patients reported satisfaction with the surgery and there was a statistically significant improvement in all describing signs and symptoms of GERD-SI (*p* < 0.001). We examined the reasons for the negative answers. The reason for not recommending or re-undergoing surgery was the persistence or the recurrence of symptoms. Although patients undergoing surgery sign an informed consent, which clearly states that the operation does not give 100% certainty that the symptoms will disappear, being in the minority is a cause for dissatisfaction. Beenen et al. investigated the problem [18]. They analyzed the patient postoperative satisfaction 18 months after LNF, asking questions about satisfaction, recommendations, and symptoms. Postoperative heartburn and regurgitation were highly predictive of patient dissatisfaction (*p* = 0.0002, *p* = 0.0003, respectively). Moreover, the lack of necessity to administer PPIs was associated with patient satisfaction (*p* = 0.003), which is similarly consistent with our observations. 

In the literature, we found an interesting study on the impact of mental disorders on patient satisfaction [19]. Holcomb et al. compared patients with depression and anxiety to a population without mental illness. While postoperative satisfaction did not differ between the depression and control groups (76% vs. 71%, *p* = 0.55), patients with anxiety had significantly lower satisfaction rates between the surgery compared to controls 40% vs. 71%, *p* = 0.01). Kessing et al. demonstrated that patients suffering from anxiety are characterized by an exaggeration of preoperative and postoperative symptoms [20]. These studies suggest that patients with anxiety disorders should be carefully examined when qualifying for surgery. For this, we need a place for an interdisciplinary team of psychiatrists and psychologists. Patients should also be made aware that in the case of comorbidities, satisfaction with the surgery may be reduced. Unfortunately, we do not have data on mental disorders in our patients, but we plan to expand research on this topic in the near future.

The information in this study is important for several reasons. All patients in our series underwent LNF in one department by the same team of surgeons, which makes this group homogenous in terms of analysis. The study is based on surgeries from 7 years ago, following a standardized technique that keeps this work up to date. Moreover, the data were collected prospectively, and the mean follow-up time is 50 months (21.2–76.3 ± 16.6 months), which makes it a long-term study. This study may help physicians and patients advise considering surgery as a treatment for GERD.

The limitations of the study are the small sample size and subjectivity in description of symptoms. The patients did not plan the pH measurement or other imaging to determine objective indicators of recurrence. Additionally, we did not report any mental disorders of patients that may have an impact on patients’ satisfaction and selection for the surgery.

## 5. Conclusions

LNF is a good treatment for GERD. According to our study, 78.4% of patients reported satisfaction with the surgery, would recommend it to their relatives, and would be open to accessing the surgery again with the knowledge they now have.

## Figures and Tables

**Table 1:** Patients’ characteristics. (±standard deviation; BMI—body mass index, Preop.—preoperative; HH—hiatal hernia; and LA—Los Angeles).

		N	%
Age range (average) (years)	18–80 (50.2 ± 15)		
BMI range (average) (kg/m^2^)	17.7–35.6 (25.9 ± 3.5)		
Follow up range (average) (months)	21.2–76.3 (50 ± 16.6)		
Gender			
	Female	49	44.1
	Male	62	55.9
Preop. Gastroscopy			
	HH, no esophagitis	14	12.6%
	Esophagitis	97	87.4%
LA Classification			
	LA-A	24	24.7%
	LA-B	62	63.9%
	LA-C	9	9.3%
	LA-D	2	2.1%
Early complications			
	No	106	95.5
	Yes	5	4.5
PPIs after surgery			
	No	94	84.7
	Yes	17	15.3
Recurrence			
	No	87	78.4
	Yes	24	21.6

**Table 2:** Median and interquartile range (IQR) of GERD-IS before and after the surgery. (1—daily; 2—often; 3—sometimes; and 4—never).

Questions	Before LNF		After LNF		*p*
	Median	25–75% IQR	Median	25–75% IQR	
How often have you had pain in your chest or behind the breastbone?	2	1–3	4	3–4	<0.001
How often have you had a burning sensation in your chest or behind the breastbone?	2	1–3	4	3–4	<0.001
How often have you had regurgitation or an acid taste in your mouth?	2	1–3	4	3–4	<0.001
How often have you had pain or burning in your upper stomach?	3	2–4	4	4–4	<0.001
How often have you had a sore throat or hoarseness that is related to your heartburn or acid reflux?	3	2–4	4	4–4	<0.001
How often have you had difficulty getting a good night’s sleep because of your symptoms?	3	2–4	4	4–4	<0.001
How often have your symptoms prevented you from eating or drinking any of the foods you like?	2	2–3	4	3–4	<0.001
How frequently have your symptoms kept you from being fully productive in your job or daily activities?	2	1–3	4	4–4	<0.001
How often do you take additional medication other than what the physician old you to take (such as Maalox, Alusal, Manti)?	3	1–4	4	4–4	<0.001

**Table 3:** Patients answers to “yes or no” questions.

	Q1. Would You Recommend Surgery to Your Relatives?					Q2. Would You Undergo Surgery Again with the Knowledge You Have Now?				
	Yes		No			Yes		No		
	N	%	N	%	*p*	N	%	N	%	*p*
Complications										
Yes	2	50	2	50	0.04	2	50	2	50	0.06
No	85	87	13	13		86	85	15	15	
Recurrence										
Yes	6	35	11	65	<0.001	8	40	12	60	<0.001
No	81	95	4	5		80	94	5	6	
PPIs after surgery										
Yes	6	43	8	57	<0.001	7	47	8	53	<0.001
No	81	92	7	8		81	90	9	10	
Age [years]										
≤65	71	87	11	13	0.75	72	87	11	13	0.11
>65	16	80	4	20		16	73	6	27	

## Data Availability

The data presented in this study are available on request from the corresponding author.
